# Probing Pedogenetic Imprints and Functional Properties of Moroccan Clayey Materials Through FFC NMR Relaxometry

**DOI:** 10.1002/mrc.70015

**Published:** 2025-07-17

**Authors:** Paola Bambina, Calogero Librici, Ettore Madonia, Francesco Lanero, Roberta Bertani, Paolo Sgarbossa, Manal Monsif, Delia Francesca Chillura Martino, Paolo Lo Meo, Pellegrino Conte

**Affiliations:** ^1^ Department of Agricultural, Food and Forest Sciences University of Palermo Palermo Italy; ^2^ Department of Industrial Engineering University of Padova Padova Italy; ^3^ Department of Biological, Chemical and Pharmaceutical Sciences and Technologies University of Palermo Palermo Italy

**Keywords:** clay, FFC ^1^H‐NMR relaxometry, micro‐scale hydrological connectivity, NMR, pedogenesis, porosity, soil

## Abstract

Understanding how soil formation processes influence the microstructure and the dynamic behavior of clay‐rich materials is essential for both pedological interpretation and technological assessment. In this study, we applied fast field cycling nuclear magnetic resonance (FFC NMR) relaxometry to investigate the microstructural heterogeneity of Moroccan clays developed under diverse pedogenetic conditions. Nuclear magnetic relaxation dispersion (NMRD) profiles were processed using a model‐free inversion algorithm to retrieve the distribution of correlation times. The latter provides a phenomenological mapping of proton–surface interactions across distinct dynamic domains. Complementary indicators of micro‐scale hydrological connectivity were, then, computed from the T₁ distributions, integrating both structural (SCI) and functional (FCI) heterogeneity. While the former indicates the breadth of molecular environments experienced by water across the system, the latter captures the dynamic contrast between fast‐ and slow‐relaxing populations associated with variations in surface accessibility and magnetic heterogeneity. The results showed that the clay sample from Khemisset exhibited the greatest relaxation heterogeneity, consistent with advanced pedogenetic reorganization related to redox‐driven redistribution of paramagnetic metals. In contrast, the clay samples from Berrechid and Tiflet displayed a more ordered architecture and lower magnetic heterogeneity, reflecting earlier‐stage pedogenetic development. This study demonstrated that FFC NMR relaxometry reveals the microstructural memory encoded into water dynamics, offering a powerful tool to infer the pedogenetic pathways leading to soil formation. Beyond its relevance for pedological studies, the method also offers valuable insights into the technological behavior of clays, supporting the selection of raw materials for industrial purposes based on their microstructural properties.

## Graphical abstract

Fast Field Cycling NMR relaxometry reveals how pedogenetic pathways imprint distinct dynamic signatures in Moroccan clays, shaping their microstructural heterogeneity and technological properties.

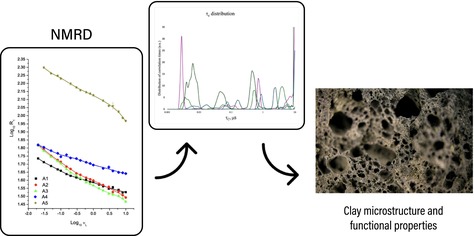

## 1 Introduction

Clays are the primary raw materials used in the production of ceramic goods, including floor and wall tiles, sanitary ware, and earthenware. The quality of these products depends on multiple factors throughout the production chain, starting with the identification and exploitation of suitable clayey deposits [1]. These clay resources derive from soils that have developed through prolonged chemical, physical, and biological transformations, beginning with the weathering of the parent material and modulated over time by pedogenetic processes [2]. Pedogenesis (i.e., soil genesis and diagenesis) progressively alters the mineralogical composition, the textural organization, and the reactivity of the soil matrix, leaving a persistent imprint on its microstructure. Processes such as eluviation/illuviation (i.e., translocation and accumulation of fine particles in deeper horizons), vertisolization (formation of shrink–swell structures due to smectitic clay dynamics), carbonatation (precipitation of secondary carbonates), and redoximorphism (formation of mottles and Fe–Mn translocation under alternating redox conditions) reorganize the pore network by modifying aggregation patterns, blocking or opening conduits, thus altering the spatial distribution and mobility of interstitial water [3, 4, 5]. For instance, lessivage (i.e., clay eluviation) tends to enhance microporosity by obstructing interparticle voids [6], while carbonatation promotes pore occlusion and surface smoothing through the precipitation of secondary carbonates along ped faces and pore walls. Under alternating redox conditions, iron mobilization and reprecipitation produce oxide coatings and cementing bridges that further narrow pore throats and limit water mobility [7]. Beyond the spatial reconfiguration of the pore network, pedogenetic transformations also modify the magnetic properties of the soil. Indeed, oxidation–reduction dynamics drive the spatial redistribution of paramagnetic transition metals, such as Fe, Mn, Co, and Ni. These elements, depending on whether they are incorporated into phyllosilicate lattices or precipitated as discrete oxides, give rise to magnetically heterogeneous microenvironments with distinct magnetic properties. Therefore, soil microstructure reflects the interplay between pore architecture and magnetic heterogeneity, both of which are tightly connected to their pedogenetic history. Standard analytical techniques such as fluid‐flow measurements, gravimetric sorption analysis or mercury intrusion porosimetry aim at assessing the macroscopic behavior of porous materials [8], but tend to overlook the microscopic features that govern molecular‐scale interactions, such as surface chemistry, pore connectivity, and the spatial distribution of relaxation‐active sites. To probe the microstructural characteristics of porous materials at the nanoscale, fast field cycling nuclear magnetic resonance (FFC NMR) relaxometry has emerged as a powerful, non‐destructive technique, providing unique insights into nuclear spin dynamics across multiple timescales (from picoseconds to microseconds) [9]. FFC NMR relaxometry offers a detailed fingerprint of the interaction between mobile proton‐bearing fluids with solid porous systems by measuring how rapidly proton spins return to their equilibrium state after being perturbed into a non‐equilibrium condition. This process, known as longitudinal, or spin–lattice, relaxation is quantified by the relaxation time T₁. This is the time constant that describes the recovery of the longitudinal magnetization and depends on how efficiently protons exchange energy with their surroundings. The recovery of the longitudinal magnetization depends on the strength of magnetic dipole–dipole interactions between pairs of spins [10]. In natural complex systems such as soils, these dipolar couplings are established between the water molecules of the saturating soil solution and solid surfaces, particularly in spatially restricted domains where molecular motion is limited. When water resides within the pore network, its translational and rotational motions are hindered by frequent collisions with mineral surfaces. These restrictions enhance local magnetic fluctuations at the Larmor frequency, thereby increasing the efficiency of dipole–dipole relaxation mechanisms [8]. In nanoporous domains, such as interlayer spaces, edge sites, and interparticle voids formed by aggregated clay particles, water experiences a high density of surface interactions per unit volume, leading to short longitudinal relaxation times (T₁). Conversely, in mesoporous domains, where the distance from the solid interface is greater and molecular motion is less constrained, water molecules experience fewer surface collisions and reduced dipolar coupling, resulting in longer T₁ values and a lower overall relaxation efficiency. However, the most effective dipolar interactions responsible for spin–lattice relaxation in soils arise from the coupling between water protons and nearby paramagnetic centers, mainly Fe^3+^, Mn^2+^, Co^2+^, and Ni^2+^. These unpaired‐electron‐bearing ions generate fluctuating local magnetic fields that match the proton Larmor frequency, thereby enhancing the spectral density of dipolar interactions and markedly increasing the probability of energy exchange between nuclear spins and the lattice [11]. The magnitude of this enhancement depends not only on the total concentration of paramagnetic species, but also on their distribution and accessibility within the pore architecture [12]. Indeed, paramagnetic ions in soils can occur in two main configurations: (i) structurally incorporated within the octahedral sheets of phyllosilicates, such as smectites, chlorites, and illites, or (ii) precipitated as discrete oxide or hydroxide phases coating mineral surfaces. The impact of these ions on proton relaxation depends on their spatial accessibility and distribution. In minerals like nontronite and some Fe‐rich illites, paramagnetic cations (e.g., Fe^3+^) are unevenly dispersed within the clay matrix, generating strong internal magnetic field gradients and enhancing surface relaxivity, which leads to shorter relaxation times [12]. Similarly, crystalline iron oxides such as goethite, when precipitated at or near pore surfaces, can act as efficient relaxation centers due to their high magnetic susceptibility and surface accessibility, further intensifying dipolar interactions. Conversely, in laminated or compositionally homogeneous matrices, paramagnetic species are less accessible to mobile water, resulting in weaker relaxation effects [13]. In Fe‐poor clays such as kaolinite, where paramagnetic centers are scarce or absent, proton relaxation is less efficient and T₁ values are consequently longer. The interaction between a proton and a nearby paramagnetic center is several orders of magnitude stronger than proton–proton dipolar coupling at equivalent distances. As a result, spin–lattice relaxation in soils is predominantly governed by the concentration, spatial distribution, and accessibility of paramagnetic ions, rather than by pore geometry alone. However, the effect of paramagnetic centers on relaxation efficiency also depends on how long water protons remain in their vicinity. This interaction timescale is quantified by the dynamic correlation time (τ_c_), which describes how long a proton retains memory of its local magnetic environment before its motion becomes uncorrelated [14]. Longer τ_c_ values indicate a more persistent interaction with nearby magnetic centers, enhancing dipolar coupling and thus accelerating spin–lattice relaxation, which results in shorter T₁ values. FFC NMR relaxometry allows for the measurement of these correlation times, enabling a detailed characterization of molecular dynamics within heterogeneous porous materials such as soils. Therefore, the accurate interpretation of NMR relaxometry data of soils requires an integrated understanding of mineralogy, topological distribution of paramagnetic centers, and pore architecture, all reflecting the pedogenetic history of the soil. Several studies have demonstrated the efficacy of FFC NMR relaxometry in investigating a variety of porous materials, including cement‐based materials [11, 15], rocks [16], soils [9, 17], biochar [18, 19, 20], and synthetic nanosponges [21]. However, to the best of our knowledge, FFC NMR relaxometry has never been explicitly applied to support pedogenetic interpretation while simultaneously assessing the technological potential of soil‐derived materials. This study aims to fill that gap by interpreting FFC relaxometric data through a pedological lens. Specifically, we applied FFC NMR relaxometry to five clay‐rich soils from three northwestern Moroccan regions (Berrechid, Tiflet, and Khemisset) with the aim of identifying the relaxation features related to pedogenetic processes. Building upon the mineralogical and chemical proxies reported by Monsif et al. [22], we also explored how microstructural features are related to the functional suitability of clayey materials. This work provides, for the first time, a framework integrating NMR relaxometry into the analysis of soil pedogenesis, even in the absence of direct morphological observations.

## 2 Materials and Methods

### 2.1 Clayey Deposits

Clay samples derived from five soils located in three distinct areas of northwestern Morocco (i.e., Berrechid, Tiflet, and Khemisset) were provided by the Super Cerame Kenitra (SCK) ceramic industry (Kenitra, Morocco). The samples from Berrechid were labelled A1, A2, and A3, while samples A4 and A5 originated from Tiflet and Khemisset, respectively. These regions are widely recognized for their extensive clay deposits, which have long been exploited for ceramic production. The selected materials were chosen for being representative of the local clay mineral assemblages typically used in industrial applications. The Berrechid deposits (33°08′17.72″ N, 7°25′48.73″ W) lie within a basin underlain by folded Paleozoic shales and argillites. The latter are overlain by an Infra‐Cenomanian to Cenomano‐Turonian sequence and capped by Pliocene to Quaternary silts, gravels, and clays [23]. The Tiflet region (33°54′24.46″ N, 6°15′49.23″ W) is underlain by Lower Ordovician siliciclastic and carbonate rocks, including shales, sandstones, and dolostones. These are overlain by Upper Silurian–Lower Devonian black shales, microconglomerates, and turbiditic limestone. The Khemisset deposits (33°46′15.77″ N, 6°07′26.76″ W) developed on Paleozoic shales and fine‐grained sandstones. These are unconformably overlain by Pliocene to Quaternary fluvio‐lacustrine sediments, composed of sands, silts, and gravels.

### 2.2 Mineralogical and Chemical–Physical Features of Clay Samples

The mineralogical and chemical–physical properties of the clay samples are reported in Monsif et al. [22]. Samples A1–A3 exhibited a mineralogical assemblage dominated by primary silicates such as quartz and muscovite, accompanied by substantial proportions of 2:1 phyllosilicates including illite, high‐charge smectites, and interstratified smectite‐illite phases with random to partially ordered stacking (referred to as S‐I R0 and S‐I R1, respectively). These latter consist of alternating 2:1 layers of illite and smectite within the same crystallite. Their structural arrangement provides key information on the transformation pathways of phyllosilicates. In S‐I R0 configurations, the stacking sequence of illite and smectite layers is completely random, reflecting early stages of mineral transformation. In contrast, S‐I R1 ordering denotes a partially regular alternation of layers, suggesting progressive illitization (i.e., the diagenetic or pedogenetic transformation of smectite into illite through the fixation of K^+^ ions in the interlayer spaces, typically under conditions of low water activity and elevated temperature or during progressive soil development) [24]. Minor quantities of vermiculite and amorphous phases are also present, along with appreciable amounts of kaolinite. This mixed mineralogy reflects an incipient to moderate stage of mineral transformation, where inherited components coexist with pedogenetically altered phases. From a chemical perspective, the samples are characterized by relatively high SiO_2_ and Al_2_O_3_ concentrations, moderate Fe_2_O_3_ contents, and a base‐rich geochemical signature, with moderate CaO and MgO. These features are indicative of low‐intensity leaching under buffered pH conditions. Notably, crystalline Fe oxides, such as goethite, are absent, despite the measurable Fe_2_O_3_ content. This suggests that Fe is predominantly structurally integrated within the octahedral sheets of phyllosilicates, rather than occurring as discrete oxyhydroxide phases. The lack of crystalline Fe oxides is consistent with pedogenetic systems in which redox fluctuations are limited and Fe mobilization and precipitation have not occurred. Low concentrations of trace paramagnetic ions, such as Mn^2+^, Co^2+^, and Ni^2+^ were detected. Particle‐size distribution data revealed a dominant silt fraction (96–98%), with very low clay content (< 2%) and minor sand. Specific surface area (SSA) ranged from 18.6 to 21.4 m^2^ g^−1^. Pore size distribution was centered around 3.8 nm, pointing to a well‐developed, homogeneous mesoporous framework. This likely arose from the aggregation of smectitic and interstratified clay particles into lamellar domains. Although the presence of smectitic components might suggest incipient shrink–swell activity, the low clay content indicates that true vertisolization (i.e., pedogenetic process characterized by the formation of deep cracks and slickensides due to repeated shrink–swell cycles in smectite‐rich soils) cannot occur. The studied samples can be rather interpreted as belonging to Cambisols, namely moderately developed soils showing initial signs of chemical–physical alteration [25]. The mineral assemblage of sample A4 from the Tiflet region is dominated by interstratified illite–smectite minerals with random stacking (I–S R0) and by the presence of calcite and dolomite. Also, minor quantities of kaolinite and chlorite were observed. This mineralogical assemblage points to a moderate weathering under buffered, base‐rich conditions. The chemical composition supports this interpretation: the sample displays high concentrations of CaO and MgO, along with moderate to low SiO_2_, Al_2_O_3_, and Fe_2_O_3_ concentrations. The absence of crystalline Fe oxides (e.g., goethite) indicates that Fe is mostly retained within clay mineral structures. The low concentrations of trace paramagnetic elements suggest a reduced overall density of magnetic centers. From a physical standpoint, the particle size distribution reveals a silty‐loam texture, with 78% of silt, 12.5% of sand, and less than 1% of clay. The SSA of 20.6 m^2^ g^−1^ and the pore size distribution centered at around 3.7 nm indicate a well‐developed homogeneous mesoporous framework. The total pore volume (0.039 cm^3^ g^−1^) is comparable to samples A1–A3. These features point to a material belonging to a moderately developed soil consistent with a carbonate‐rich Cambisol. Sample A5 was characterized by a complex assemblage dominated by interstratified illite–smectite minerals with random stacking (I–S R0) and by quartz, alongside significant amounts of muscovite, illite. Crystalline goethite was also detected, highlighting oxidative weathering and the development of redox‐sensitive pedogenetic features. The presence of both Fe_2_O_3_ and goethite suggests that Fe is partitioned between structural sites of phyllosilicates and discrete secondary oxides. The chemical profile reveals high SiO_2_, moderate Al_2_O_3_ and Fe_2_O_3_, and low MgO and CaO. This suggests advanced leaching under neutral (to acidic) conditions. Also, high concentrations of paramagnetic trace elements, such as V, Cr, Co, and Ni, were observed, likely structurally integrated within metal‐bearing mineral phases. From a physical standpoint, A5 exhibits a predominantly silty texture (94.5% of silt, 1.6% of clay), coupled with very high SSA (35.5 m^2^ g^−1^). Pore size analysis revealed a broad and asymmetric mesopore distribution, lacking a distinct dominant peak. The profile shows a gradual increase in pore volume across the 4–20 nm range, with minor inflections around ~3.8 nm and a shoulder between 15 and 25 nm. This is indicative of a disordered, heterogeneous mesoporous network. The total pore volume was also notably higher (0.081 cm^3^ g^−1^) as compared to the other samples. These features suggest the coexistence of interparticle mesopores and larger, irregular intra‐aggregate voids, likely originating from microstructural disaggregation and mineral reorganization. Such patterns are consistent with redox‐driven pedogenetic processes (i.e., redoximorphism), which foster iron mobilization, redistribution, and reprecipitation as oxides. This mineralogical complexity, together with a diversified and disordered pore system, suggests that the clay material derives from a pedogenetically evolved soil developed under prolonged and alternating redox conditions. SoilGrids predictions further support these pedogenetic interpretations [26].

### 2.3 The FFC NMR Experiment

The clay samples were dried at 60 °C for 48 h to obtain the bulk materials (Monsif et al., 2019) [22]. Then, 1 g of each sample was suspended in 3 mL of MilliQ grade water (electrical resistivity 18.2 MΩ cm at 25 °C) and transferred into the NMR tubes to perform the FFC NMR analysis. The experiment was carried out by means of a Stelar SmarTracer Fast‐Field‐Cycling Relaxometer (Stelar s.r.l., Mede, PV–Italy) at the constant temperature of 25 °C, as described by Conte and Ferro [27]. Three fundamental steps are generally recognized in a FFC NMR relaxometry experiment: the preparation, the relaxation, and the acquisition steps. When a magnetic field of *polarization* (B_POL_) is applied for a fixed period of time (T_POL_), the preparation step is indicated as pre‐polarization and a pre‐polarized (PP) sequence is achieved. When no B_POL_ is applied, the preparation step is referred to as non‐polarization and a non‐polarized (NP) sequence is achieved. After the preparation, the magnetic field is switched towards a new magnetic field, called *relaxation* field (B_RLX_). This is applied for an arrayed period of time (τ), ranging in the interval [0, τ_RLX_], during which the magnetization intensity reaches a new equilibrium condition. To generate the observable, a third magnetic field, called *acquisition* field (B_ACQ_), is applied while a ^1^H 90° pulse is held for a fixed period of time. Then, the free induction decay (FID) is acquired. In this study, the B_RLX_ was varied along the proton Larmor frequency (*ν*_*L*_) range of 0.02–10 MHz. When the B_RLX_ varied in the ν_L_ range 0.02–3 MHz, a B_POL_ of 10 MHz was applied for a period of around four times the estimated *T*_*1*_ at this frequency (PP sequence). When the B_RLX_ varied in the *ν*_*L*_ range 4–10 MHz, the B_POL_ was null, and a non‐polarized (NP) sequence was applied. The time of application of each B_RLX_ (τ) varied on 32 logarithmic spaced time sets and was adjusted at every relaxation field in order to optimize the recording of the decay/recovery curves. The FIDs were recorded after applying a ^1^H 90° pulse of 5.5 μs at an acquisition field (B_ACQ_) of 7.2 MHz. The field‐switching time was 3 ms and the spectrometer dead time was 15 μs. One thousand points were sampled in a time domain of 100 μs. The recycle delay was 7.2 s.

#### 2.3.1 The FFC NMR Data Elaboration

The FIDs provide the recovery and the decay curves for the NP and the PP sequences, respectively. Both the curves describe the evolution of the ^1^H longitudinal magnetization [M(τ)] for each B_RLX_. The regression analysis of the recovery/decay curves provides the most probable value of *T*_*1*_ for each B_RLX_. These curves are described by the sum of N exponential functions [28]: *N* = 1 when homogeneous molecular fluctuations are present in simple chemical systems (such as in pure solvents); *N* > 1 when dealing with complex systems, where different dynamic domains are simultaneously present. When the different components of the molecular dynamics are described by very close longitudinal relaxation times, the most probable distribution of *T*_*1*_ (D(*T*_*1*_)) is obtained by applying the regularized inverse Laplace transform. This is represented by the following equations for the PP and NP sequences, respectively:

$$M\left(\tau \right)=\int_{T_{1\min}}^{T_{1\max}}D\left(T_{1}\right)\;\exp[-\left(\frac{\tau }{T_{1}}\right)D\left(T_{1}\right)+\sigma$$

$$M\left(\tau \right)=\int_{T_{1\min}}^{T_{1\max}}D\left(T_{1}\right)\;\left\{1\right.-\exp[-\left.\left(\frac{\tau }{T_{1}}\right)\right\}D\left(T_{1}\right)+\sigma$$

*T*_*1*min_ and *T*_*1*max_ are the longitudinal relaxation time limits, D(*T*_*1*_) is the *T*_*1*_ distribution function, and σ is an unknown noise component. This latter makes it impossible to calculate the exact relaxation time values. Thus, the most probable *T*_*1*_ distribution is obtained by considering some constraints, such as the smoothness of the solution and the variance of the experimental data. The regularized inverse Laplace transform was performed by means of the Uniform PENalty regularization (UPEN) algorithm [30]. The resulting plot, reporting the D(*T*_*1*_) vs. the *T*_*1*_ values, is referred to as a relaxogram.

#### 2.3.2 The NMRD Profiles

The nuclear magnetic relaxation dispersion (NMRD) profiles were obtained by plotting the longitudinal relaxation rates *R₁* = 1/*T₁* as a function of the proton Larmor frequency (*ν*_*L*_) over the range of 0.02–10 MHz. These curves describe how the spin–lattice relaxation of water protons varies with magnetic field strength, thereby providing insight into the characteristic timescales of water dynamics within the different microenvironments of the porous matrix. Classical approaches to NMRD profile interpretation, such as the Bloembergen–Purcell–Pound (BPP) model [29], are effective in simple systems dominated by a single relaxation mechanism. More complex approaches, like those by Halle et al. [30] and Kruk et al. [31], extend the analysis to heterogeneous systems by treating the NMRD curve as a sum of Lorentzian functions, each associated with a distinct motional regime. However, these models require prior assumptions about the number of relaxation components, which may introduce biases when applied to structurally complex materials such as soils. To overcome these limitations, the experimental NMRD data were elaborated by using the heuristic algorithm proposed by Lo Meo et al. [14]. This model‐free, data‐driven method treats the porous system as a continuum of dynamic domains, each characterized by a specific correlation time (τ_c_). This represents the average timescale of molecular reorientation or translational motion. The heuristic method builds upon the general formalism by Kruk et al. [31], where the relaxation rate is expressed as follows:

$$R_{1\;\left(\nu_{L}\right)}=R_{0}+R^{\mathrm{HH}}+R^{\mathrm{NH}}$$

*R*_*0*_ is the offset keeping into account frequency‐independent fast molecular motions (τ_c_ < 1 ns), *R* ^*NH*^ describes the occurrence of the quadrupolar dips (when present), and the *R* ^*HH*^ represents the ^1^H‐^1^H relaxation described as the sum of three BPP‐like components.

Lo Meo et al. proposed replacing the discrete sum of the three BPP‐like components in R^HH^ with a continuous integral over a distribution of correlation times:

$$R^{\mathrm{HH}}=\int_{0}^{\infty }\left[\left(\frac{\tau_{c}}{1+{\omega \tau }_{c}}\right)+\left(\frac{4\;\tau_{c}}{1+4\;{\left({\omega \tau }_{c}\right)}^{2}}\right)\right]f^{*}\tau_{c}d\tau_{c}$$

Here, ω is the Larmor angular frequency, and $f^{*}\tau_{c}=C^{\mathrm{HH}}\cdot f\left(\tau_{c}\right)$ incorporates the dipolar coupling constant C^HH^ which depends on the average internuclear distance. To solve the inverse integral transform, a Monte Carlo algorithm is used. This ensures mathematical stability, reduces noise‐induced artifacts, and estimates the uncertainty associated with each τ_c_ value.

To facilitate interpretation, the correlation times are converted to a logarithmic space as λ_c_ = Log_10_(τ_c_). The transformed distribution l*(λ_*c*_ *)* is then computed according to l*(λ_*c*_ *)* = [10^λc^·f*(10^λc^)]^1/2^.

Although the model‐free inversion approach avoids a priori assumptions about the number of relaxation components, the deconvolution of logarithmically scaled data may occasionally produce artefactual peaks that are regularly spaced by a factor of ~10 in τ_c_. To mitigate this risk, we applied a Monte Carlo resampling procedure to evaluate the stability and reproducibility of the extracted components. Only peaks that consistently emerged across multiple inversion runs and whose amplitudes exceeded the noise threshold were retained. The function l*(λ_*c*_ *)* is built by means of the ModelFreeFFC graphical interface developed in Matlab by Bortolotti et al. [32]. The resulting plots provide a visual representation of the distribution of dynamic domains.

#### 2.3.3 The Micro‐Scale Hydrological Connectivity

The relaxograms, reporting the distribution D(T₁) as a function of relaxation time T_1_, were used to calculate the micro‐scale hydrological connectivity (μ‐HC), following the approach proposed by Conte et al. [27]. This concept draws inspiration from the classical definition of hydrological connectivity in soil science, which refers to the continuity of soil water‐conducting pathways at the macroscopic scale. In the present study, this notion is reinterpreted through the lens of NMR relaxometry, with the aim of capturing the continuity, accessibility, and functional diversity of water domains at the molecular level. Micro‐scale hydrological connectivity (μ‐HC) quantifies how effectively water molecules interact with the internal pore network of the clayey soil matrix. It reflects both the structural complexity of the porous system and the magnetic heterogeneity of the solid phase, which together shape the diversity of relaxation behaviors arising from water–matrix interactions. It is conceptualized as the sum of two complementary descriptors: the structural connectivity index (SCI) and the functional connectivity index (FCI). The SCI captures the degree of heterogeneity of water‐accessible microenvironments. High SCI values indicate that water molecules experience a wide range of dynamic environments, from domains where weak water–matrix interactions result in slow proton relaxation, to regions where relaxation is markedly enhanced by strong dipolar coupling with solid surfaces or paramagnetic centers. This variability reflects the combined structural and magnetic complexity of the porous system. The FCI, on the other hand, quantifies the dynamic contrast within the sample by measuring the span between the fastest and slowest relaxing water populations. It captures variations in water–matrix interactions driven by differences in surface chemistry, accessibility of relaxation‐active sites, and local magnetic heterogeneity. A high FCI reflects marked dynamic heterogeneity, with coexisting microenvironments characterized by strongly divergent relaxation behaviors. Together, SCI and FCI offer an integrated perspective on how microstructural variability and magnetic complexity jointly shape water dynamics in porous media. Both indices derive from the relaxograms by computing the non‐exceeding empirical cumulative frequency function, Φ(T₁). This function is obtained by associating to each T₁ value the ratio A_T₁/_A_*tot*_. A_T₁_ is the area delimitated by the relaxogram curve, the *T*_*1*_ axis, and the ordinate value corresponding to the *T*_*1*_ value, while A_*tot*_ is the total area under the relaxogram [33]. Two key reference points are extracted from Φ(T₁) and are indicated with T_A_ and T_B_, respectively. T_A_ is defined as the T₁ value where Φ(T₁) = 0.01 and represents the boundary of the fastest relaxing 1% of the water population, typically confined in strongly interacting environments (such as interlayer spaces of phyllosilicates and crystalline defects). T_B_ is defined as the T₁ value where Φ(T₁) = 0.99 and represents the upper boundary of the slowest‐relaxing 1% associated with water residing in mesopores with limited surface interaction and short residence times. Mathematically, these are given by:

$$\Phi \left(T_{A}\right)=\frac{1}{A_{\mathrm{tot}}}\;\int_{0}^{T_{A}}D\left(T_{1}\right)dT_{1}=0.01$$

$$\Phi \left(T_{B}\right)=\frac{1}{A_{\mathrm{tot}}}\;\int_{0}^{T_{B}}D\left(T_{1}\right)dT_{1}=0.99$$

SCI is calculated as the coefficient of variation (CV) of T₁ values in the central range of the Φ(T₁) distribution, i.e., between Φ(T₁) = 0.01 and Φ(T₁) = 0.99. FCI is calculated as the ratio between T_B_ and T_A_, capturing the extent of dynamic contrast between slow and fast‐relaxing water populations.

The selection of thresholds Φ(T₁) = 0.01 and Φ(T₁) = 0.99 was made to encompass the full spectrum of observable relaxation behavior, while minimizing the influence of outliers and instrumental noise. These percentile‐based thresholds target the outermost tails of the distribution, thereby ensuring the inclusion of both strongly and weakly interactive water populations. This approach enhances the sensitivity of SCI and FCI to subtle variations in soil microstructure. Similar cutoffs have been effectively applied in previous relaxometric studies [34] to define dynamic boundaries. Sensitivity tests (not shown) using alternative thresholds (e.g., 0.05 and 0.95) confirmed that the general connectivity trends were preserved, although with reduced dynamic contrast. This supports the adoption of the 0.01–0.99 interval as a robust criterion for evaluating connectivity across heterogeneous porous systems.

## 3 Results and Discussion

### 3.1 The NMRD Profiles of Moroccan Clay Samples

**FIGURE 1:**
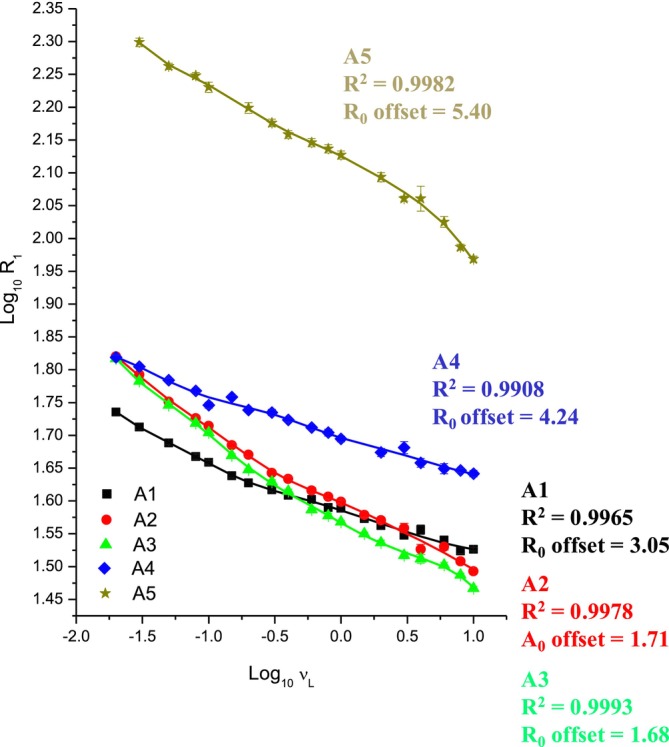
NMRD dispersion curves of the five clay samples analyzed by means of FFC NMR analysis. The regression analysis was performed by means of the heuristic algorithm proposed by Lo Meo et al.

The NMRD profiles of the five Moroccan clay samples were obtained by plotting the longitudinal relaxation rates (R₁ = 1/T₁) as a function of the proton Larmor frequency over the range of 0.02–10 MHz. These curves were analyzed using the heuristic, model‐free algorithm proposed by Lo Meo et al. [14]. This is a data‐driven method that reconstructs the distribution of the correlation times (τ_c_) in a complex system without imposing a priori the number of dynamic components. The excellent agreement between the experimental and fitted curves (*R* ^2^ > 0.99) confirms the ability of the method to capture the intrinsic complexity of water–matrix interactions in complex porous systems such as soils (Figure 1). *R* ^2^ denotes the coefficient of determination, which quantifies the proportion of variance in the experimental data that is captured by the fitted model. Values close to 1 indicate an excellent fit, confirming the model's reliability. The offset term R₀, also shown in Figure 1, represents the frequency‐independent component of the relaxation rate. This contribution typically arises from ultra‐fast molecular motions typically associated with bulk‐like water dynamics [14]. However, in hydrated clay systems, this baseline is not solely associated with bulk water. Instead, it also reflects the presence of solvated paramagnetic ions, either dissolved in the pore solution or loosely adsorbed onto mineral surfaces, which induce local magnetic field fluctuations that accelerate proton relaxation independently of the Larmor frequency.

In this study, the R₀ values ranged from 1.68 to 5.40 s^−1^, far exceeding the expected R₀ for pure bulk water (~ 0.3 s^−1^) [35]. These elevated values indicate that proton mobility is strongly influenced by the presence of paramagnetic species and the chemically complex nature of the aqueous phase. It should also be noted that T₁ components shorter than 1.5 ms, potentially arising from highly confined environments or regions with intense magnetic susceptibility, fall below the temporal resolution of the present FFC‐NMR setup. Although not captured here due to instrumental limitations (T₁ < 1.5 ms), such ultra‐fast components are expected to account for a minor fraction of the total proton population. The dominant relaxation behavior, and hence the extracted correlation time regimes, reflect the most prevalent dynamic environments in the system. These environments, associated with mesoporous domains, govern the bulk NMRD response and are thus robustly captured within the FFC‐NMR detection window. Nevertheless, the existence of unresolved ultra‐fast regimes remains plausible and could be addressed through complementary high‐resolution NMR methods in future studies. The shape and slope of the NMRD profiles (Figure 1) reflect the degree of field dependence of the longitudinal relaxation rate (R₁), which in turn is influenced by the local molecular environment of water. Steeper profiles, as observed in sample A5, indicate pronounced frequency dependence and are typically associated with water molecules experiencing restricted mobility due to strong interactions with solid surfaces or localized paramagnetic centers. This suggests the presence of slow dynamic regimes and heterogeneous relaxation environments. Conversely, flatter profiles, such as those of samples A1–A3, exhibit weaker dispersion and suggest faster molecular dynamics, consistent with mesoporous domains and less efficient coupling with relaxation‐active sites.

### 3.2 Distribution of Correlation Times as Indicative of Different Dynamic Regimes

**FIGURE 2:**
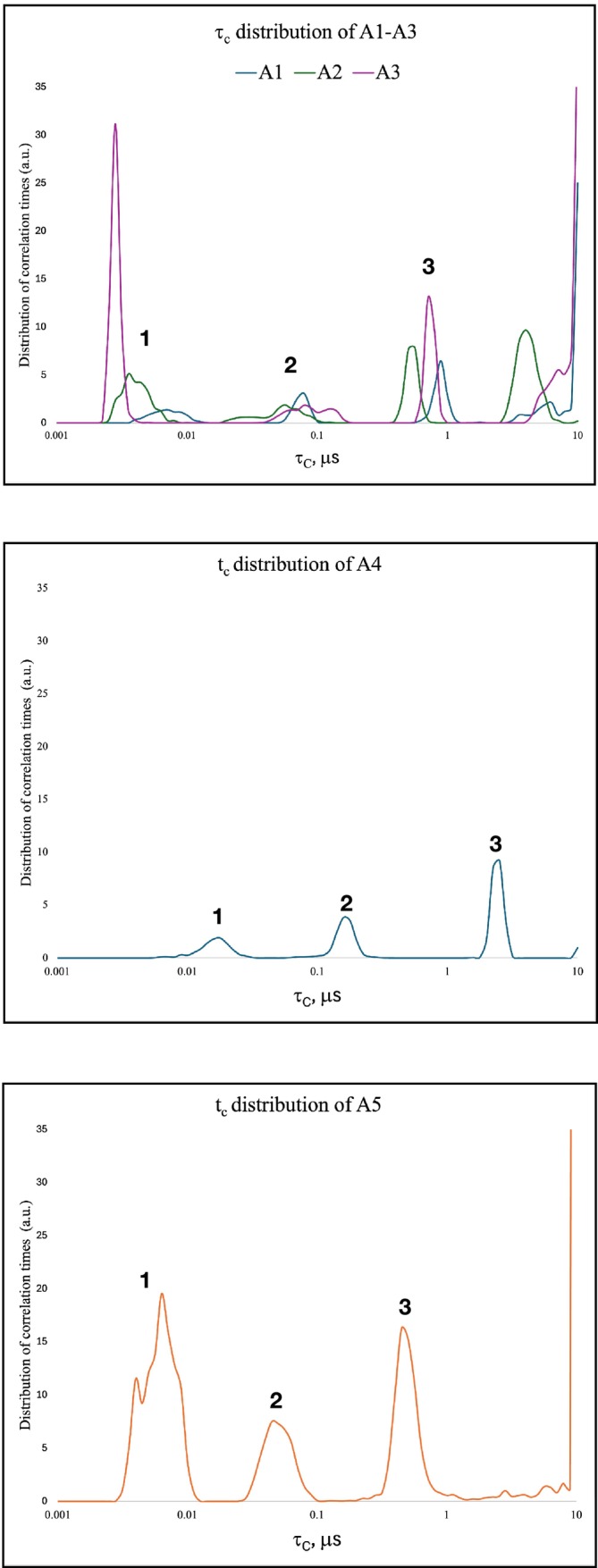
Correlation time (τ_c_) distributions for samples A1–A5. Numbers identify the three main dynamic components: (1) fast, (2) intermediate, and (3) slow regimes. The interpretation of each domain is summarized in Table 1.

The τ_c_ distributions resulting from the heuristic inversion of the NMRD profiles are reported in Figure 2. They revealed the presence of three dynamic regimes in all samples, corresponding to proton populations characterized by different molecular mobility. These dynamic regimes have been indicated as fast, intermediate, and slow regimes (Table 1) and reflect the increasing degrees of rotational and translational restriction experienced by water molecules within different microenvironments. The correlation time τ_c_ characterizes the average duration over which a molecule maintains a given orientation or position before reorienting or translating [32]. It is strongly influenced by both the geometric features of the pore network and the strength of local interactions with the surrounding solid matrix. Short τ_c_ values indicate high molecular mobility, typically associated with large pores, low surface affinity, and minimal magnetic interaction. Conversely, longer τ_c_ values reflect slower molecular dynamics due to strong interactions with the solid surfaces or spatial confinement within small pores, where water mobility is restricted and residence times are prolonged. This behavior is governed by the distribution and accessibility of paramagnetic species embedded in the solid matrix and by the architecture of the pore system. As outlined in Section 2.2. of Materials and Methods, the concentration and spatial distribution of paramagnetic centers vary markedly across the five samples. In samples A1–A3, paramagnetic ions (primarily Fe^3+^, Mn^2+^, and Ni^2+^) are structurally integrated within phyllosilicate layers, thus resulting in a relatively homogeneous magnetic system. This configuration promotes moderate and spatially uniform magnetic interactions, leading to narrowly distributed τ_c_ values. Sample A4 shows a lower overall concentration of paramagnetic elements and lacks discrete Fe oxide phases, suggesting limited contribution from strongly magnetic centers. However, the presence of secondary carbonates (i.e., calcite and dolomite) forming coatings or cementing bridges along pore surfaces may hinder water mobility by stabilizing surface hydration layers and narrowing pore throats. This progressive reduction in pore size, driven by pedogenic carbonatation, limits diffusion pathways and enhances spatial confinement. These microstructural features may increase τ_c_ even in the absence of paramagnetic relaxation enhancement. In contrast, sample A5 contains high concentrations of paramagnetic species including Fe^3+^, Cr^3+^, Co^2+^, Ni^2+^, and V^3+^. These ions occur both as structurally bound components within the lattice of phyllosilicates and as discrete phases, including crystalline Fe‐oxides, such as goethite. This mixed occurrence creates a strong magnetic heterogeneity. Water molecules exhibit reduced mobility and extended residence near paramagnetic sites, leading to broader and asymmetric τ_c_ distributions. The pore size distribution reported in Monsif et al. showed that samples A1–A4 exhibit an ordered pore architecture with only one sharp mesopore peak centered at ~3.8 nm. Differently, sample A5 shows a multimodal distribution without a dominant peak, indicative of a disordered and irregular mesoporous framework. In light of the above, the fast component in samples A1–A3 (τ_c_ ≈ 0.003–0.007 μs) is attributed to water molecules residing in inter‐aggregate mesopores (i.e., pores located between clay particle aggregates, typically formed by the spatial arrangement of lamellar phyllosilicates), where molecular mobility remains high and the proximity to paramagnetic sites is limited. The intermediate component (τ_c_ ≈ 0.06–0.09 μs) reflects water in intra‐aggregate mesopores (i.e., pores located within individual soil aggregates, typically formed by the stacking and packing of phyllosilicate lamellae), which create a moderately confined pore network with limited accessibility and enhanced water–surface interactions. This regime likely corresponds to the sharp mesopore peak at ~3.8 nm (Monsif et al., 2019). The slow component (τ_c_ ≈ 0.5–0.9 μs) corresponds to water in less accessible microdomains, such as collapsed interlayers or compacted aggregates, where molecular motion is severely slowed due to geometric occlusion or prolonged interaction with paramagnetic centers. Sample A4 exhibits a τ_c_ distribution systematically shifted towards longer correlation times across all dynamic regimes, indicating a generalized slowdown of molecular mobility throughout the pore network. The fast component (τ_c_ ≈ 0.02 μs) likely corresponds to water residing in mesopores partially occluded by carbonate coatings or cementing bridges, which reduce effective pore throat size and stabilize hydration layers at the mineral–solution interface, thereby slowing down reorientational dynamics. The intermediate component (τ_c_ ≈ 0.16 μs) is attributed to water located within intra‐aggregate mesopores structured by illite‐ and kaolinite‐dominated domains. These pores exhibit limited internal connectivity and moderate confinement, especially in kaolinitic areas where the platy habit and lower swelling capacity constrain the accessibility of internal surfaces. The slow component (τ_c_ ≈ 2.6 μs) reflects water molecules located in microporous or occluded domains, including collapsed interlayer spaces or compacted microaggregates, where restricted diffusion and prolonged interaction with solid surfaces dominate. The absence of discrete Fe oxide phases combined with the presence of secondary carbonates supports the hypothesis of a partially restructured pore network. Sample A5 displays the broadest and most asymmetric τ_c_ distribution. The fast component (τ_c_ ≈ 0.006 μs) exhibits a multimodal signature, pointing to the coexistence of mesoporous domains with distinct degrees of structural and chemical organization. These include loosely arranged regions associated with amorphous or poorly crystalline phases, where water mobility is relatively unimpeded, as well as more ordered mesopores spatially adjacent to clusters of transition metal oxides (e.g., goethite), which can locally influence the motion of nearby water molecules through surface interactions and short‐range structuring of the aqueous phase [28]. The intermediate component (τ_c_ ≈ 0.05 μs) is broader and reflects the presence of mesoporous domains that lack a regular geometric framework. This dynamic behavior is consistent with the internal disaggregation and reassembly of clay particles, possibly enhanced by the accumulation of Fe‐rich secondary minerals and amorphous phases. These materials introduce spatial irregularity and increase the complexity of the pore space, limiting water reorientation without fully immobilizing it. The slow component (τ_c_ ≈ 0.5 μs) is indicative of water molecules residing in restricted domains with low physical connectivity, such as occluded micropores, compacted microaggregates, or zones stabilized by mineral crusts and coatings. These microdomains likely result from advanced weathering, redox cycling, and secondary mineral precipitation, which collectively reconfigure the clay matrix and impose long‐lived constraints on molecular motion. It is important to highlight that the τ_c_ components identified through the model‐free inversion are not interpreted as fixed relaxation constants associated with discrete pore types or specific mechanistic pathways. Rather, they represent a phenomenological mapping of distinct dynamic regimes emerging from the interplay between pore dimension and geometry and spatial distribution of paramagnetic centers. This interpretative framework allowed us to relate the observed τ_c_ domains to functional microenvironments, without invoking rigid structural or theoretical constraints. Nevertheless, the τ_c_ patterns observed here are consistent with findings related to other porous systems, including cement‐based materials [11, 15], rocks [16], soils [17, 28, 34], biochar [18, 20, 33], and synthetic nanosponges [21], where short correlation times are generally linked to water residing in relatively open domains with weak spatial constraints, while longer correlation times typically reflect water confined within microporous or structurally complex regions, where molecular mobility is significantly hindered by narrow geometries, reduced connectivity, and surface‐induced ordering. The present study moves beyond a purely descriptive approach and establishes a link between the observed dynamic relaxation regimes to pedogenetic pathways. Indeed, the interpretation of distinct τ_c_ patterns in relation to clay aggregation, carbonate formation, and redox‐driven metals redistribution revealed how molecular‐scale water dynamics retain a functional memory of soil‐forming processes.

**TABLE 1:** Interpretation of the correlation time (τ_c_) components identified in the FFC NMR relaxation spectra of samples A1–A5. Each component is associated with specific dynamic domains and dominant proton–matrix interaction mechanisms.

Sample	Component	τ_c_ (μs)	Dynamic domains	Dominant mechanism
**A1–A3**	Fast	0.003–0.007	Inter‐aggregate mesopores (i.e., pores between clay aggregates)	High mobility, low relaxivity due to limited paramagnetic accessibility
	Intermediate	0.06–0.09	Intra‐aggregate mesopores (formed by stacked lamellar clays)	Moderate confinement, Fe^3+^ in illite–smectite structure
	Slow	0.5–0.9	Micropores and collapsed interlayers	Long residence near embedded paramagnetic centers
**A4**	Fast	~0.02	Carbonate‐coated mesopores	Stabilized hydration layers, restricted diffusion pathways
	Intermediate	~0.16	Intra‐aggregate mesopores (illite–kaolinite)	Low connectivity, limited paramagnetic relaxation
	Slow	~2.6	Occluded micropores or compacted domains	Physical confinement, prolonged residence time
**A5**	Fast	~0.006 (multimodal)	Disordered mesopores with variable magnetic environment	Strong heterogeneity, surface‐accessible Fe oxides and trace metals
	Intermediate	~0.05	Amorphous intra‐aggregate domains	High surface relaxivity from poorly crystalline Fe‐rich phases
	Slow	~0.5	Micropores with goethite and Fe oxides	Enhanced dipolar coupling, localized magnetic fields

### 3.3 Longitudinal Relaxation behavior and Water Dynamics

**FIGURE 3:**
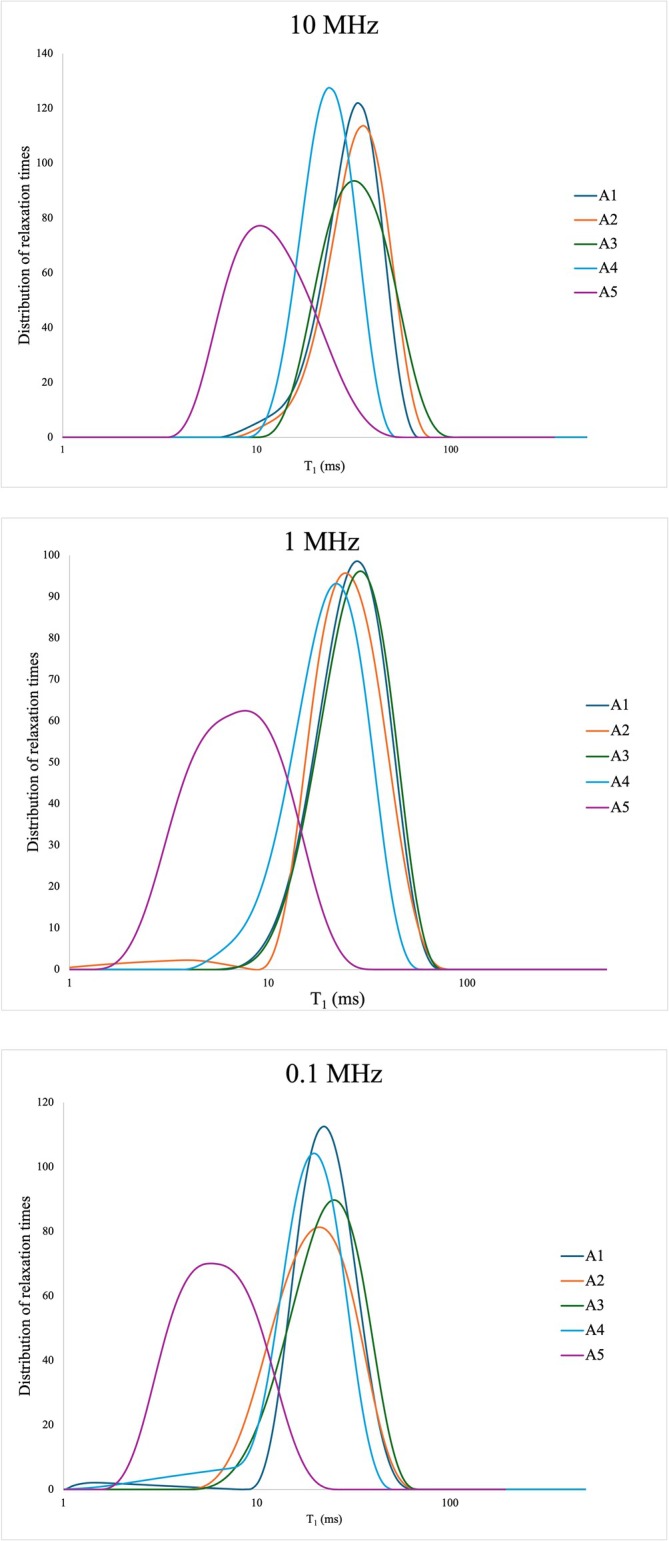
Relaxograms of the clay samples at three representative proton Larmor frequencies, namely 10 MHz, 1 MHz, and 0.1 MHz.

The longitudinal relaxation times (T₁) measured across the five Moroccan clay samples span a broad range, from approximately 1.5 ms to over 8000 ms within the investigated Larmor frequency interval (0.02–10 MHz). This wide variability reflects the coexistence of multiple relaxation mechanisms acting over different spatial and temporal scales. In porous materials such as clays, spin–lattice relaxation (T₁) is primarily governed by the dipolar interactions between water protons and nearby magnetic moments. These interactions are modulated by two key factors: the presence of unpaired electrons in paramagnetic species (e.g., Fe^3+^, Mn^2+^, Co^2+^) and the dynamics of water molecules as they diffuse and reorient within the porous matrix, described by the correlation time τ_c_ [10]. Short T₁ values arise when water molecules dwell near paramagnetic centers long enough (τ_c_ in the microsecond range) to enable efficient dipolar relaxation. Conversely, fast‐diffusing water in less interactive environments (e.g., large mesopores or bulk‐like domains) exhibits longer T₁ values due to inefficient coupling. When paramagnetic ions are structurally integrated into phyllosilicates or are present as finely dispersed oxides, they generate local magnetic fields that enhance spin relaxation, especially when proton correlation times (τ_c_) are long enough to ensure sustained proximity to the magnetic sites. These effects are clearly reflected in the T₁ relaxograms. The relaxograms of the clay samples at three representative Larmor frequencies are shown in Figure 3. Sample A5 exhibits consistently shorter T₁ values across all frequencies, indicating the co‐occurrence of strong spatial proximity to paramagnetic centers and motional regimes favorable to dipolar energy exchange. The high concentration of Fe^3+^, V^3+^, Cr^3+^, Co^2+^, and Ni^2+^, present both as structural components of phyllosilicates and as surface‐accessible oxides (e.g., goethite), generates local magnetic field gradients that intensify relaxation. The irregular pore geometry and high degree of magnetic heterogeneity further promote dynamic contrast and broaden the relaxation spectrum.

By contrast, samples A1–A3 showed longer T₁ values consistent with limited paramagnetic enhancement. Paramagnetic ions in these samples are mostly structurally bound within the clay lattice, resulting in a more homogeneous magnetic landscape and reduced local field fluctuations. The mesoporous structure centered at ~3.8 nm allows for restricted but not strongly confined diffusion, further contributing to the observed slower relaxation. Sample A4 exhibits intermediate behavior: Although poor in discrete Fe oxides, it contains carbonate phases (calcite, dolomite) that may reduce internal porosity and pore interconnectivity. These structural effects likely increase water–surface residence time and partially compensate for the low paramagnetic content, leading to moderately shortened T₁ values. Overall, these observations confirm that FFC NMR relaxometry is sensitive to both geometric confinement and magnetic enhancement.

### 3.4 Micro‐Scale Hydrological Connectivity (μ‐HC)

**FIGURE 4:**
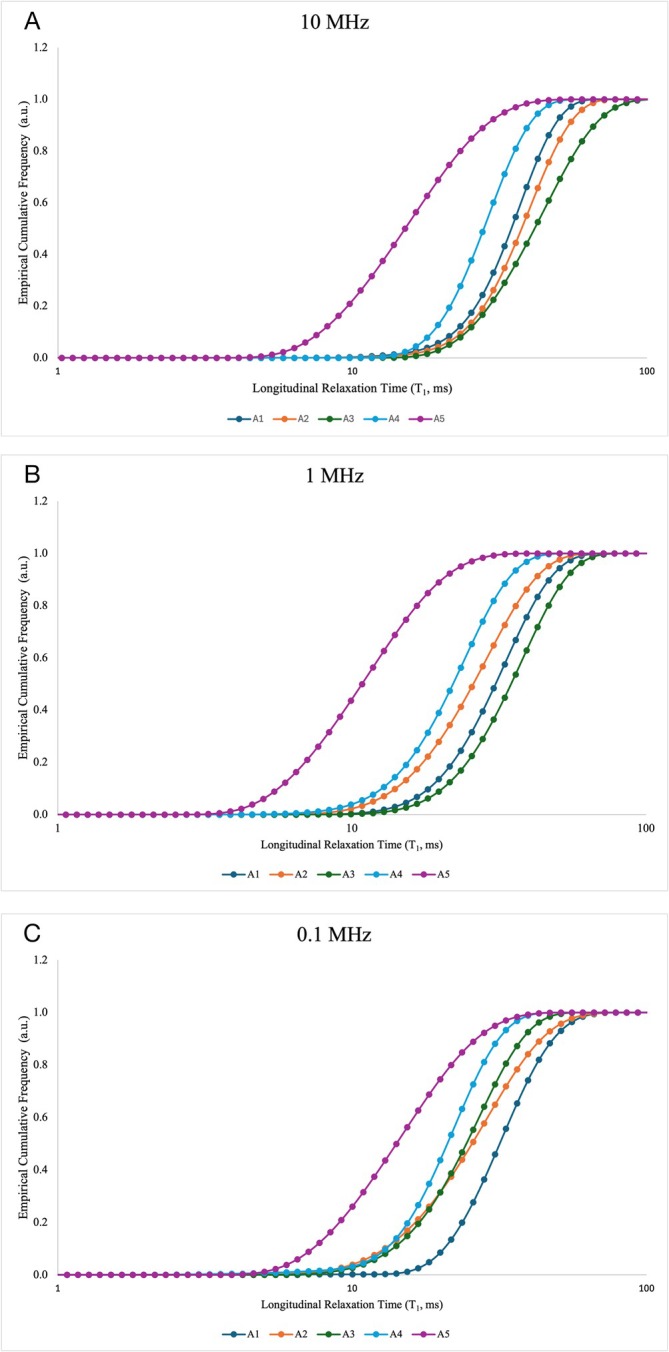
Non‐exceeding empirical cumulative frequency Φ(*T*_*1*_) of the clay samples at three representative proton Larmor frequencies, namely 10 MHz, 1 MHz, and 0.1 MHz.

Figure 4 shows the non‐exceeding empirical cumulative frequency curves Φ(T₁) derived from the relaxation time distributions of the five Moroccan clay samples at three representative proton Larmor frequencies (i.e., 10 MHz, 1 MHz, and 0.1 MHz).

**FIGURE 5:**
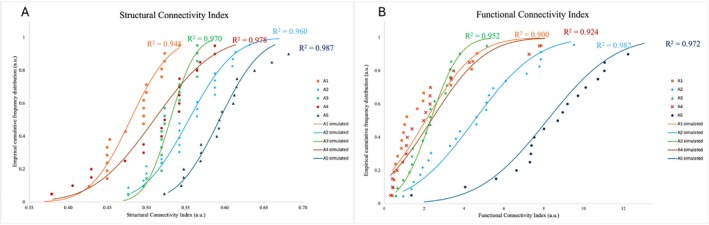
The SCI and FCI plotted vs. the empirical cumulative frequency distribution Φ(*T*_*1*_). The curves were S‐shaped and normally distributed. Hence, the distributions of the two parameters can be exhaustively described by the mean ± standard deviation indicated in Table 1.

These curves provide a non‐parametric, integral representation of the proton relaxation dynamics, allowing the extraction of two key thresholds, T_A_ and T_B_, that delimit the dynamic heterogeneity of the system. Specifically, T_A_ corresponds to Φ(T₁) = 0.01 and represents the lower threshold of the fast‐relaxing 1% of the water populations, typically associated with highly constrained environments. Conversely, T_B_, corresponding to Φ(T₁) = 0.99, captures the upper limit of the slowest‐relaxing 1% of water protons, reflecting domains where molecular motion is least restricted. The central limb of the Φ(T₁) curve, between T_A_ and T_B_, reflects the diversity of dynamic regimes experienced by water molecules within the pore network. A wider interval indicates greater heterogeneity in local environments and water–matrix interactions, suggesting the coexistence of domains with markedly different relaxation behaviors. Conversely, a narrower interval points to a more homogeneous system, where water protons experience relatively uniform molecular constraints and relaxation conditions. Sample A5 displays the widest Φ(T₁) range and the most gradual slope in the central segment of the curve, reflecting marked microstructural heterogeneity and a broad spectrum of water–matrix interactions. This behavior aligns with its complex mineralogical and chemical composition: A5 contains significant amounts of goethite and various paramagnetic ions (e.g., Fe^3+^, Co^2+^, Ni^2+^), which enhance spin–lattice relaxation by increasing surface relaxivity and generating heterogeneous local magnetic fields. In this sample, the observed relaxometric response is not solely governed by pore size distribution but also by the spatial variability in magnetic susceptibility arising from the uneven distribution of paramagnetic species. In contrast, samples A1, A2, and A3 display steeper Φ(T₁) curves and narrower T_A_–T_B_ intervals, indicative of reduced dynamic heterogeneity. This behavior is consistent with a more homogeneous internal architecture dominated by mesoporous domains generated by the lamellar aggregation of 2:1 phyllosilicates, such as illite and smectite. Although these clays contain moderate amounts of Fe_2_O_3_, the absence of discrete Fe oxide phases and the structural incorporation of Fe^3+^ within the clay lattice contribute to a more uniform distribution of magnetic relaxation centers. Consequently, the relaxation environment is more consistent across the pore system, leading to less dispersed relaxation behavior. Sample A4 exhibits an intermediate behavior, with a slightly broader Φ(T₁) distribution compared to A1–A3. This behavior can be primarily attributed to the presence of secondary carbonates (e.g., calcite and dolomite), which form coatings and cementing bridges along pore surfaces. These microstructural features restrict diffusion pathways and stabilize surface hydration layers, leading to longer correlation times and a moderate increase in relaxation heterogeneity. In the absence of discrete Fe oxide phases, the observed dynamics are mainly governed by carbonate‐induced structural effects rather than by magnetic heterogeneity. To objectively quantify the internal relaxation heterogeneity, two indices, derived from the empirical cumulative distribution function Φ(T₁), were computed: the Structural Connectivity Index (SCI) and the FCI, according to Conte et al. (2020) [27]. Their sum was defined as micro‐scale hydrological connectivity (μ‐HC). The SCI quantifies the statistical dispersion of longitudinal relaxation times between the 1^st^ and 99^th^ percentiles of the empirical cumulative distribution Φ(T₁). It serves as a proxy for the degree of heterogeneity in the local environments sampled by water molecules, encompassing both pore structure variability and the spatial distribution of relaxation‐active sites. The FCI, defined as the ratio T_B_/T_A_, captures the dynamic contrast within the system by expressing the span between the most and least efficiently relaxing water populations. Together, these metrics provide complementary insights: while SCI emphasizes the spread and complexity of local interactions, FCI reflects the range of relaxation regimes, integrating contributions from both geometrical confinement and paramagnetic enhancement [36]. These indices are dimensionless and facilitate the comparison of relaxation behavior across samples. The normal distribution of SCI and FCI values across the frequency range (Figure 5) justifies their expression as mean ± standard deviation (Table 2). Sample A5 exhibited the highest values for all three metrics (SCI = 0.59 ± 0.04, FCI = 8 ± 3, μ‐HC = 9 ± 3), indicating a strongly heterogeneous water population shaped by both complex pore architecture and pronounced magnetic heterogeneity. The elevated SCI and FCI values highlight the cumulative impact of advanced redoximorphic processes, such as iron redistribution, goethite neoformation, and kaolinitization, which generate spatially variable surface relaxivity landscapes. These transformations result in broad, multimodal T₁ distributions, reflecting coexisting dynamic regimes with markedly different water–matrix interactions. Samples A1–A4 exhibited consistently lower values of the indices indicative of more uniform relaxation behaviors and moderate dynamic contrast within the pore system. This trend reflects more conservative pedogenetic trajectories, where physical aggregation of 2:1 phyllosilicates and limited mineralogical differentiation result in relatively homogeneous pore networks and a lower degree of magnetic complexity. In these clays, the distribution of paramagnetic species is more uniform, with Fe^3+^ structurally incorporated within phyllosilicate sheets. These findings underscore that micro‐scale hydrological connectivity (μ‐HC), as captured by FFC NMR relaxometry, is not solely determined by pore geometry but is intimately linked to pedogenetic transformations that modulate the magnetic environment. Processes such as iron mobilization and reprecipitation, redox cycling, and clay mineral transformations alter not only the chemical composition of the matrix but also the spatial distribution, density, and accessibility of paramagnetic relaxation centers. These modifications shape the relaxation landscape at the nanoscopic scale, generating functional heterogeneity that reflects the legacy of soil‐forming processes. Hence, μ‐HC, when interpreted alongside τ_c_ distributions and mineralogical data, emerges as a process‐sensitive proxy capable of tracing the imprint of pedogenesis on internal microstructure. The integration of FFC NMR relaxometry with pedological analysis reveals that pedogenesis does not merely reorganize solid phases and pore space but actively reshapes the magnetic topology encountered by water protons, ultimately modulating the dynamic behavior of the soil matrix.

**TABLE 2:** Structural connectivity index (SCI), functional connectivity index (FCI), and the microscale‐hydrological connectivity (μ‐HC) measured for the clay samples. Different Latin letters indicate significant differences (*p* < 0.05) derived by the one‐way ANOVA coupled with Tukey's HSD post hoc tests.

Clay sample	SCI				FCI				μ‐HC			
**A1**	0.48	±	0.04	a	2	±	1	a	3	±	1	a
**A2**	0.55	±	0.05	c	5	±	2	b	5	±	3	b
**A3**	0.53	±	0.03	bc	2	±	1	a	3	±	1	a
**A4**	0.51	±	0.06	ab	2	±	1	a	3	±	1	a
**A5**	0.59	±	0.04	c	8	±	3	c	9	±	3	c

### 3.5 Technological Implications of Relaxometric Behavior in Clay Materials

The relaxometric profiles of the Moroccan clays provide a functional perspective on their internal microstructure, which in turn influences their technological performance. The combination of T₁ distributions, correlation time domains, and hydrological connectivity indices offers a non‐destructive means of assessing internal heterogeneity, water–matrix interaction regimes, and potential reactivity, key factors in determining the suitability of natural clays for various industrial applications.

Samples A1–A3 (Berrechid) exhibit narrow T₁ distributions and moderately short relaxation times, consistent with water located in relatively uniform mesoporous domains formed by lamellar stacking of illite–smectite aggregates. The presence of structurally bound paramagnetic ions (Fe^3+^, Mn^2+^, Co^2+^) ensures efficient relaxation without introducing magnetic complexity. This leads to a stable and predictable dynamic response, typically associated with good plasticity and shaping behavior, properties favorable for structural ceramic products like bricks and tiles, where formability must be balanced with shrinkage control. This pattern reflects a more rigid and chemically buffered microstructure, dominated by illite, kaolinite, and carbonate phases. The reduced accessibility of paramagnetic centers and the pore‐narrowing effect of carbonate coatings likely limit both water mobility and surface reactivity. Consequently, A4 is expected to exhibit reduced plasticity but enhanced dimensional and thermal stability, making it suitable for decorative ceramics or thermally resistant applications. In contrast, sample A5 (Khemisset) displays broad T₁ distributions, short relaxation times, and a high dynamic contrast, indicative of a structurally and magnetically heterogeneous system. The coexistence of crystalline goethite with other trace paramagnetic ions creates intense local magnetic fields that accelerate proton relaxation and reveal highly reactive microenvironments. These features suggest limited workability but high chemical reactivity, making A5 potentially suitable for use in adsorption and catalysis processes, as a pozzolanic additive, or in the formulation of geopolymers and pigment‐rich materials [37]. In summary, FFC NMR relaxometry enables the functional characterization of clays beyond conventional mineralogical or textural analyses, offering insights into water–matrix interactions and internal organization that are essential for evaluating the technological performance of raw materials. The relaxometric response thus emerges as a powerful proxy for both pedogenetic complexity and technological potential, guiding the targeted valorization of these materials in diverse industrial contexts.

## 4 Conclusions

This study demonstrates the potential of fast field cycling NMR (FFC NMR) relaxometry as a powerful and non‐invasive tool to investigate the internal organization and functional behavior of natural clays shaped by pedogenetic processes. By integrating relaxometric parameters with mineralogical and physicochemical data, we were able to resolve distinct water–matrix interaction regimes and reconstruct microstructural features that are otherwise difficult to capture using conventional techniques. The NMRD‐derived correlation time distributions and longitudinal relaxation profiles provided a phenomenological mapping of water dynamics across microenvironments with varying surface chemistry, pore accessibility, and magnetic complexity. Quantitative indices such as the structural connectivity index (SCI), functional connectivity index (FCI), and μ‐HC highlighted the interplay between pore architecture and the spatial distribution of paramagnetic centers. These indicators enabled us to discriminate among contrasting pedogenetic trajectories: sample A5 (Khemisset) exhibited the highest degree of relaxometric heterogeneity, driven by redox‐induced iron redistribution, goethite neoformation, and spatially variable magnetic landscapes. In contrast, samples A1–A4 displayed more uniform relaxation behavior, consistent with mesoporous structures stabilized by illite–smectite aggregation and, in the case of A4, carbonate precipitation that restricts water mobility and narrows the pore size distribution. By capturing the molecular‐scale dynamics of water across diverse microstructural domains, FFC NMR relaxometry emerges as a sensitive proxy for both structural and magnetic heterogeneity. It not only reflects the current state of soil materials but also retains the imprint of pedogenetic transformations such as lessivage, carbonatation, and redoximorphism. These results are particularly significant in the absence of direct field observations, offering a process‐sensitive approach to infer soil development and evaluate clay resource suitability for technological applications. Overall, this work positions relaxometry as a promising interpretative framework at the interface of soil science and material technology that connects the micro‐scale organization with the legacy of soil evolution.

## Conflicts of Interest

One of the co‐authors, Prof. Pellegrino Conte, is currently serving as Guest Editor for the Special Collection “NMR Relaxometry: State of the Art and Future Perspectives” in Magnetic Resonance in Chemistry. To ensure an impartial review process, the editorial handling of this manuscript has been requested to be conducted independently by an editor who is not involved as an author. The authors declare no other conflicts of interest.

## Peer Review

The peer review history for this article is available at https://www.webofscience.com/api/gateway/wos/peer‐review/10.1002/mrc.70015.

## Data Availability

The data that support the findings of this study are available from the corresponding author upon reasonable request.
